# Neonatal Clomipramine Exposure Disrupts Epididymal Serotonin Signaling and Programs Sperm Dysfunction in Adult Rats

**DOI:** 10.3390/ijms27031535

**Published:** 2026-02-04

**Authors:** Herlinda Bonilla-Jaime, Ofelia Limón-Morales, Ernesto Rodríguez-Tobón, José Edwin Mendoza-Sánchez, David Yoab Jaimes, José Luis Cortés-Altamirano, Alfonso Alfaro-Rodríguez, Marcela Arteaga-Silva, Gilberto Pérez-Sánchez, Lenin Pavón, Edith Arenas-Rios

**Affiliations:** 1 Laboratorio de Psicobiológia Conductual, Departamento de Biología de la Reproducción, Universidad Autónoma Metropolitana-Iztapalapa, Mexico City 09340, Mexico; ofelia.limon@yahoo.com (O.L.-M.);; 2 Laboratorio de Morfofisiología y Bioquímica del Espermatozoide, Departamento de Biología de la Reproducción, Universidad Autónoma Metropolitana-Iztapalapa, Mexico City 09340, Mexico; rotoern@xanum.uam.mx (E.R.-T.);; 3 Doctorado en Biología Experimental, División de Ciencias Biologicas y de la Salud, Universidad Autónoma Metropolitana-Iztapalapa, Mexico City 09340, Mexico; jesmendoza57@gmail.com; 4 Maestría en Biología de la Reproducción Animal, División de Ciencias Biologicas y de la Salud, Universidad Autónoma Metropolitana-Iztapalapa, Mexico City 09340, Mexico; yoab_elizalde@hotmail.com; 5 Departamento de Neurociencias, Instituto Nacional de Rehabilitación, “Luis Guillermo Ibarra Ibarra”, Calzada México Xochimilco No. 289 Col., Arenal de Guadalupe, Mexico City 14389, Mexico; 6 Departamento de Quiropráctica, Universidad Estatal del Valle de Ecatepec, Ecatepec de Morelos 55210, Mexico; 7 Laboratorio de Psicoinmunología, Dirección de Investigaciones en Neurociencias del Instituto Nacional de Psiquiatría Ramón de la Fuente Muñiz, Calzada México-Xochimilco 101, Colonia San Lorenzo Huipulco, Tlalpan, Mexico City 14370, Mexico; gilberto.perez.sanchez@inprf.gob.mx (G.P.-S.); lkuriaki@inprf.gob.mx (L.P.)

**Keywords:** clomipramine, serotonin, mitochondrial activity, reactive oxygen species, serotonin transporter

## Abstract

Studies of adult depressed patients report that selective serotonin (5-HT) reuptake inhibitors (SSRIs) like clomipramine (CMI) have secondary effects on sperm quality. The epididymis possesses an autonomous serotonergic system critical for sperm maturation, whose establishment during neonatal development remains unexplored as a target for SSRI programming. We hypothesized that neonatal CMI exposure would disrupt the developing epididymal 5-HT system, leading to permanent sperm dysfunction. CMI (30 mg/kg s.c.) was administered to male rats between postnatal days 8–21. At 3 months, sperm from the epididymal cauda was evaluated, and 5-HT levels were measured in the testis, caput, and cauda epididymis. Our novel findings demonstrate that neonatal CMI exposure induces region-specific, long-term alterations in epididymal 5-HT levels (decreased in caput, increased in cauda) without affecting testicular 5-HT. This reprogramming of the local serotonergic milieu was associated with reduced sperm concentration, viability, normal morphology, and motility, alongside increased mitochondrial activity and reactive oxygen species. This study reveals, for the first time, that the epididymal serotonergic system is a key target for neonatal SSRI programming, providing a mechanistic link (altered 5-HT homeostasis) between early-life exposure and persistent sperm defects in adulthood.

## 1. Introduction

Currently, the number of people diagnosed with depressive disorder who must use this type of medication has increased, now ranging from 2 to 10%, depending on the country [1]. Also, depressive disorder in childhood and adolescence has increased in recent years [2]. Today, selective serotonin (5-HT) reuptake inhibitors (SSRI) use has increased to 25.6% among adolescents (13–25 years) and to 19.1% among older adults (≥60) [1,3,4]. Most antidepressants used today are known to produce adverse effects in men, including various forms of sexual dysfunction; 25–73% of patients treated with SSRIs such as clomipramine (CMI) experience difficulties with ejaculating in the former age group and reaching orgasm in the latter [5].

SSRIs may also affect sperm quality and fertility. In adult humans, bupropion treatment decreases sperm concentrations and motility, accompanied by increased DNA fragmentation [1]. In adult rats treated with CMI for 8 weeks, sperm quality parameters and testosterone levels are altered. Four weeks after drug withdrawal, the effects on sperm quality parameters are restored [6]. However, studies on the effects of SSRIs during critical stages of the maturation of epididymis or testis are scarce.

During the postnatal stage, developmental events occur in the reproductive system, including spermatogonial, Sertoli cell, and Leydig cell proliferation and maturation in the rat’s testes from postnatal day (PND) 1. Moreover, around PND 15–19, the blood–testis barrier (BTB) forms [7], and from PND 8–15 the epididymis elongates by 2 m, completing the coiling of the tubule and the formation of the septa that separates its segments. Later, the rat’s epididymis cells differentiate, though it is not until PND 49 that all the epididymis cells do so fully [8]; this critical developmental window represents a vulnerable period during which pharmacological interventions could have lasting, organizational effects on reproductive physiology.

The existence of a serotonergic system in the rat testis and epididymis indicates that it regulates some aspects of male reproductive function, with its highest concentration in the caput of the epididymis [9,10,11]. It has reported the presence of the limiting enzyme for 5-HT synthesis, the Tryptophan hydroxylase (TPH), the 5-HT transporter as well as 5-HT1A, 5-HT2A, and 5-HT3 receptors in both cauda cells in the epididymis and Leydig cells [12], suggesting that the existence of a local and autonomous serotonergic system in the testis and epididymis regulate some aspects of male reproductive function. In addition, the presence of TPH in the caput has been reported and it was observed that both its activity and the availability of the local 5-HT system, particularly in epididymal maturation and its long-term programming, remains poorly understood. A growing body of evidence supports the existence of a complete, autonomous serotonergic system within the epididymis, independent of neural input. This system includes the rate-limiting enzyme for 5-HT synthesis TPH, the 5-HT reuptake transporter (SERT), and functional receptors (e.g., 5-HT1A, 5-HT2A, 5-HT3, 5-HT4). This local 5-HT machinery is strategically positioned to regulate key epididymal functions. Therefore, 5-HT is likely the master regulator of epididymal physiology. Consequently, pharmacological disruption of this system during its establishment in early life could have profound and permanent consequences.

Little is known about the function of 5-HT in this organ and even less about its effects on the maturation of the epididymis. As a result, any substance that produces alterations during these critical stages of development interferes with the normal maturation processes of the Sertoli cells and the formation of the BTB may affect spermatogenesis [9], affect sperm quality, and persist in adulthood.

Here we present our central hypothesis: The neonatal window (PND 8–21) is a critical period not only for epididymal organogenesis but also for the establishment of its 5-HT signaling system. Administration of the SSRI CMI during this phase by chronically inhibiting SERT could permanently “reprogram” epididymal 5-HT homeostasis. This alteration in a key signaling system during its establishment would have long-lasting organizational consequences, compromising epididymal function and, therefore, sperm quality in adulthood. To date, no study has investigated the long-term impact of neonatal SSRI exposure on the epididymal serotonergic system and its functional correlation with sperm dysfunction.

Therefore, the main objective of this study was to evaluate the hypothesis that neonatal exposure to CMI induces persistent, region-specific alterations in 5-HT levels in the epididymis and the testis, and that these alterations constitute an underlying mechanism for the sperm quality defects observed in adulthood. Beyond describing sperm phenotypes, we sought to provide mechanistic evidence by directly linking the dysregulation of a local signaling system (5-HT) to early programmed reproductive dysfunction.

## 2. Results

**Figure 1:**
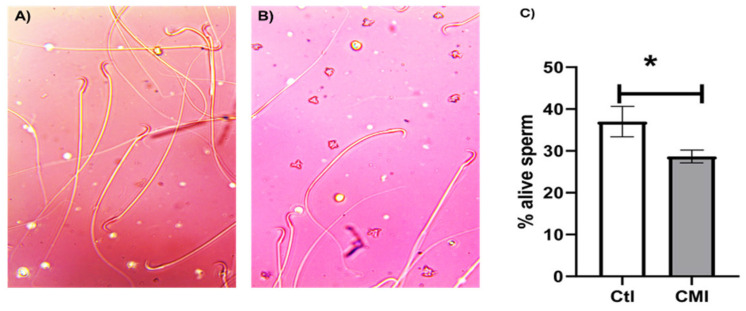
Viability of the sperm obtained from the epididymides of adult rats treated neonatally with CMI. Panel (**A**): Control spermatozoids stained with eosin/nigrosine identifying alive, unstained and dead, stained spermatozoids. Panel (**B**): CMI spermatozoids. Panel (**C**): Percentage of alive spermatozoids in the control group (Ctl) compared to the CMI-treated group. CMI treatment reduced the number of alive spermatozoids. Mean ± SEM. * *p* < 0.05. Student’s *t*-test. Bar = 10 µm.

Figure 1 shows viability of the sperm obtained from the epididymides of neonatally treated with CMI. Panel 1A is a photograph of the alive and dead spermatozoids obtained from the caudal region of the epididymis of control rats. Alive and dead sperm stained with eosin/nigrosin are visible. Panel 1B shows spermatozoids obtained from the caudal region of the epididymis of CMI-treated rats and Panel 1C shows the percentage of alive spermatozoids. Neonatal treatment with CMI significantly reduced (*p* < 0.05) the number of alive spermatozoids compared to the control group (29 ± 1% vs. 37 ± 3%).

**Figure 2:**
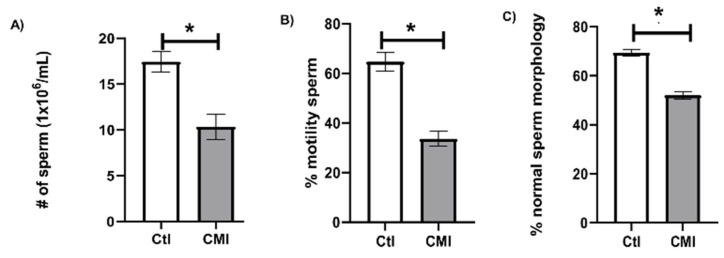
Sperm concentration (**A**) (million/mL), percentage of mobility (**B**), and percentage of spermatozoids with normal morphology (**C**) in samples obtained from the cauda of the epididymides of rat pups born to adult female rats treated neonatally with CMI. Sperm concentrations were lower in the CMI-treated group than Ctl. Likewise, the sperm from the CMI-treated cubs had lower indices of motility and normal morphology. Mean ± SEM. * *p* < 0.05. Student’s *t*-test.

With respect to sperm concentration (Figure 2A), expressed in millions/mL, observations showed that the CMI group presented a lower concentration (10 × 10^6^ ± 1) than the control group (17 × 10^6^ ± 1). Figure 2B shows the percentage of total motility in the spermatozoids. The CMI-treated group presented a lower percentage of total motility (*p* < 0.05) (33 ± 3%) than controls (64 ± 3%). Figure 2C presents the percentage of spermatozoids with normal morphology. The CMI group had a significant reduction in the percentage of normal spermatozoids (*p* < 0.05) compared to the control group (69 ± 1% vs. 52 ± 1%).

**Figure 3:**
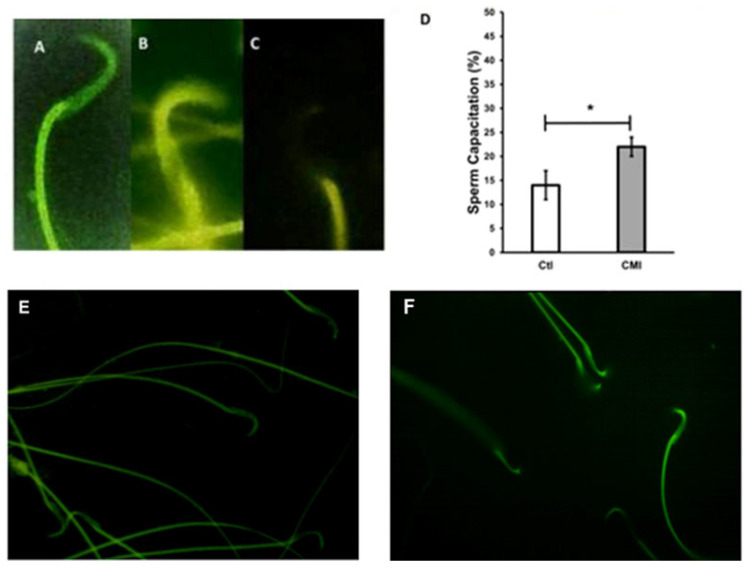
Sperm capacitation. Spermatozoids stained with CTC. Panel (**A**): Uncapacitated spermatozoid (completely stained). Panel (**B**): Capacitated spermatozoid (acrosome and principal piece stained). Panel (**C**): spermatozoid with the acrosomal reaction (without the acrosome). Panel (**E**): spermatozoid from the control group. Panel (**F**): spermatozoid from the group treated with CMI. Panel (**D**): shows the sum of the percentages of net sperm capacitation (percentage of capacitation at end time—time 0) of the rat sperm from the caudal region of the epididymis. Control group compared to the CMI-treated group. Mean ± SEM. * *p* < 0.05. Student’s *t*-test.

Figure 3 displays the three patterns found after chlortetracycline staining (CTC). Panel A shows an uncapacitated spermatozoid, Panel B a capacitated spermatozoid, and Panel C a spermatozoid with the acrosomal reaction. Greater capacitation is observed in the sperm obtained from the samples of the CMI-treated group (Figure 3D) (22 ± 2%) compared to those from the control rats (14 ± 3%).

**Figure 4:**
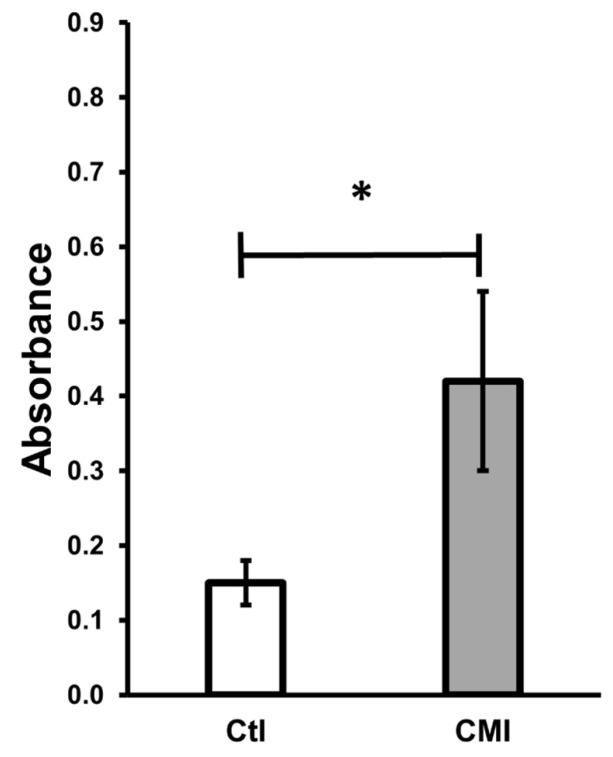
Mitochondrial activity determined after 6 h of capacitation. The group treated with CMI presented greater activity than Ctl. Mean ± SEM. * *p* < 0.05. Student’s *t*-test.

Figure 4 presents the mitochondrial activity determined by the MTT technique using sperm from the cauda of rats treated neonatally with CMI. Greater mitochondrial activity is observed in the sperm obtained from the CMI-treated rats (0.42 ± 0.12) compared to the control group (0.15 ± 0.03).

**Figure 5:**
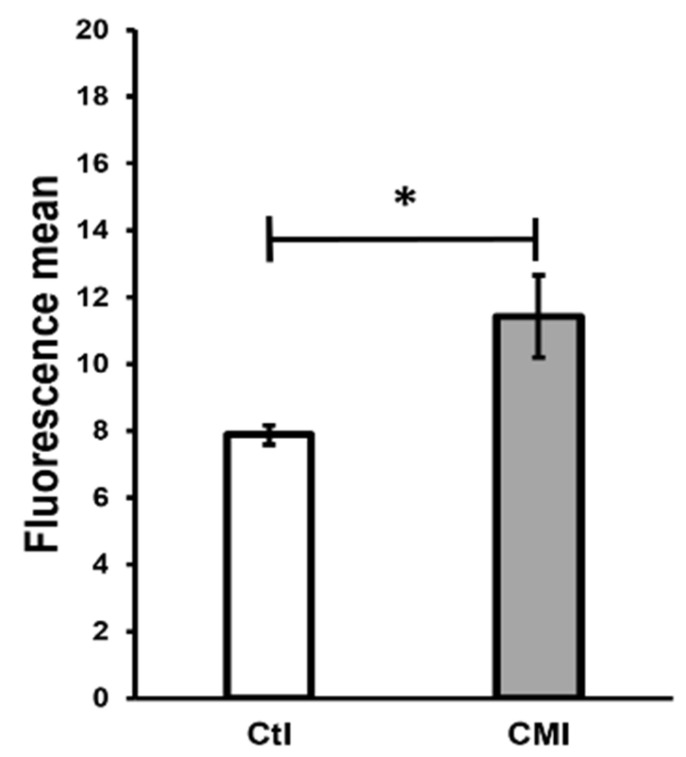
The effect of adding 2′,7′-dichlorofluorescin diacetate (DCF) expressed in the fluorescence medium in sperm from the caudal region of the epididymides of pups treated neonatally with CMI, showing an increase in ROS. Mean ± SEM. * *p* < 0.05. Student’s *t*-test.

Figure 5 shows the effect of adding 2′,7′-dichlorofluorescin diacetate (DCF) to the sperm from rats treated neonatally with CMI. The spermatozoids from those rats presented a significant increase (*p* < 0.05) in the concentration of ROS (mean fluorescence = 11.42 ± 1) with respect to the controls (7.88 ± 0.5).

**Figure 6:**
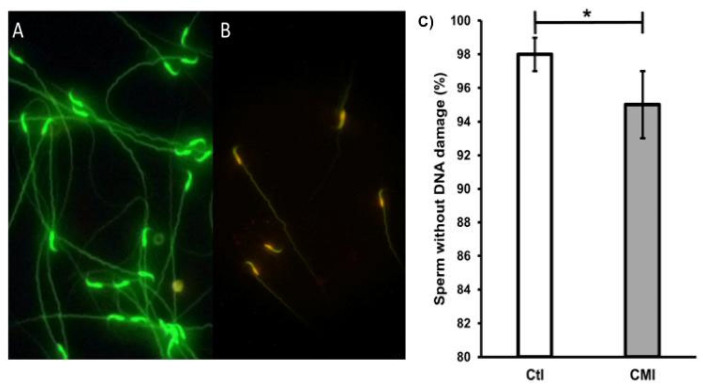
Photomicrography of rat sperm from the caudal region of the epididymis without (**A**) and with DNA damage (**B**) and Panel (**C**) shows an increase in the percentage of DNA damage in the CMI-treated group. Mean ± SEM. * *p* < 0.05. Student’s *t*-test.

Figure 6 shows the spermatozoids with (Figure 6A) and without DNA damage (Figure 6B), determined by the acridine-orange technique. The graph in Figure 6C presents the percentage of sperm without DNA damage. A higher percentage of cells without damage (98 ± 1%) is seen in the control group compared to the treatment group (95 ± 2%) (*p* < 0.05, Student’s *t*-test).

**Figure 7:**
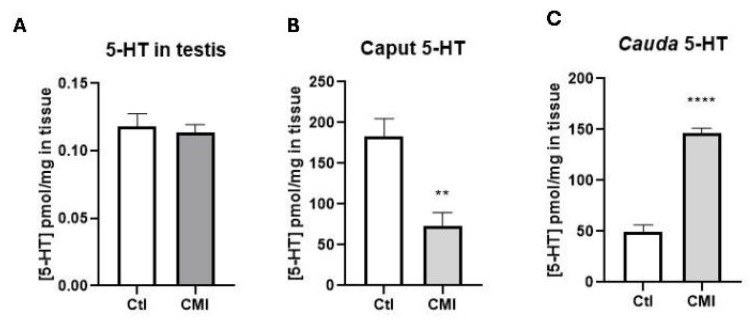
Levels of 5-HT in the testis (**A**), caput (**B**) and cauda (**C**). 5-HT levels were not modified in the testis, but 5-HT levels decreased in the group treated with CMI in the caput epididymis compared to control animals. 5-HT levels in the cauda of the CMI group were increased compared to the control group (*p* < 0.0001), as shown in Panel (**B**). Media ± EEM. ** *p* < 0.01; **** *p* < 0.0001. Student’s *t*-test.

Figure 7 shows 5-HT levels in the testis as the caput and cauda of the epididymis. Treatment with CMI produced variations in the levels of 5-HT only in epididymis, which were dependent on the specific region analyzed. 5-HT levels were not modified in the testis (Figure 7A), but 5-HT levels—measured as pmol/mg of tissue—decreased in the group treated with CMI in the caput epididymis compared to control animals (Panel B; *p* = 0.004). In contrast, the mean difference in the 5-HT concentration in the cauda of the CMI group was 96.82 pmol/g of tissue compared to the control group (*p* < 0.0001), as shown in Panel B.

## 3. Discussion

The most significant and novel finding of this study is the demonstration that transient neonatal exposure to CMI permanently reprograms the epididymal serotonergic system in a region-specific manner, and that this alteration constitutes a plausible mechanism for the sperm dysfunction observed in adulthood. Unlike the reversible effects reported in adult treatments [6], our model reveals a persistent organizational dissociation between testicular and epididymal 5-HT regulation. The fact that 5-HT decreases in the caput but increases in the cauda suggests that neonatal SERT inhibition does not cause a simple global deficit but rather disrupts the spatial gradients or fine-tuning mechanisms of 5-HT that are essential for the segmental functions of the epididymis in sperm maturation, storage, and protection [13]. This regional reprogramming of 5-HT homeostasis emerges as the central conclusion, providing a mechanistic link between early pharmacological intervention and long-term reproductive toxicity.

Our results show the neonatal CMI exposure induces region-specific, long-term alterations in 5-HT levels within the epididymis (decreased in caput, increased in cauda), without affecting testicular 5-HT. Previous studies support the finding that the caput of the epididymis has the capacity to produce 5-HT locally, with the epithelial cells acting as the principal source of 5-HT synthesis. Other sources of 5-HT include neuroendocrine cells, mastocytes, and vascular cells, as well as the tubular liquid that comes from the testicles and contributes significantly to the total amount of 5-HT in the head area [11,12]. Sperm maturation occurs during transit through the epididymis, where the sperm cells interact with the unique luminal environment of each epididymal region. In the epididymis, 5-HT has been related to the release of Cl^−^, HCO_3_, Na^+^, and H^+^ ions from the epididymal epithelium to the lumen [14], and to the transport of spermatozoids through the epididymal tract [15]. Ion release influences pH regulation, thus favoring the differentiation of the microenvironments in the distinct regions of the epididymis. This promotes changes in the isoelectric point of the proteins that foster their activation/deactivation in the sperm membrane [13]. In this case, treatment with CMI lowered 5-HT levels; this could modify the microenvironment of the epididymis and its function on sperm maturation, leading to the observed alterations.

Interestingly, the concentration of 5-HT in the caput of the epididymis increased during sexual maturation, though levels of 5-hydroxyindoleacetic acid remained essentially unchanged [12]. However, observations of the adult rats treated with CMI showed a reduction of 5-HT that had repercussions for the process of sexual maturation. Tryptophan is an amino acid precursor for 5-HT synthesis, but the enzyme indoleamine 2,3-dioxygenase 1 (Ido1) competes to capture tryptophan for its transformation into linoleic acid through the kynurenine (KYN) pathway. There are reports that Ido1 is expressed constitutively only in the caput of the epididymis [16]. This enzyme increases its activity through inflammatory processes and administration of CMI, which induces oxidative stress and inflammation [6] that favor the KYN pathway, thus fostering the reduction in 5-HT in the caput of the epididymis. Broadly speaking, this decrease in 5-HT seems to affect the maturation process of the epididymis and, as a result, that of the spermatozoids as well.

Other reports, however, suggest that 5-HT acts as a contractile agent in the rat’s epididymis [14], so it plays a role in regulating luminal activity there; that is, the contents and quiescence of spermatozoids during storage in this region of the organ. In males, an increase in plasma 5-HT levels are associated with deficient sperm counts and motility [15]. In our work, an increase in 5-HT levels was observed in the cauda of the epididymis of the CMI rats, an effect possibly due to the accumulation of 5-HT from both the testicles and the caput of the epididymis that could affect sperm concentration and motility. This region-specific dysregulation of 5-HT is a striking new finding. It suggests that neonatal CMI does not merely cause a global deficit or excess but rather disrupts the precise spatial gradient or regulatory mechanisms of 5-HT within the epididymis, which may be essential for its segment-specific functions in sperm maturation, storage, and protection.

In this regard, during the first 15 PNDs, the rat’s epididymis performs a series of cell divisions until it reaches a length of almost 2 m and completes the coiling of the tubule and formation of the septa that separate its segments. Halo cells and narrow and columnar cells appear on PND 14 and 15, respectively. The latter differentiate into basal and principal cells by PND 28 [8]. The correct differentiation of the epididymis determines this organ’s main functions in adulthood, including sperm maturation. Therefore, an alteration of the delicate 5-HT signaling during this period could permanently modify epididymal functions. During the first days of postnatal life (8–20), pronounced mitotic activity occurs in the germ cells, progenitor Leydig cells, and Sertoli cells [17]. Studies have shown that SSRIs such as CMI affect voltage-gated, Na^+^, K^+^, and Ca^2+^ channels in somatic cells [18,19,20,21]. The testes contain voltage-gated channels that may play an important role in spermatogenesis and reproduction, such as the inward rectifier K^+^ [22] and pH-sensitive K^+^ channels [23]. Germ cells express channels involved in water balance and pH control, as well as voltage-gated ion channels, like Ca^2+^ and K^+^, all of which participate significantly in spermatogenesis [24,25,26]. Agents that open or block ion channels impair spermatogenesis and, consequently, fertility. SSRIs are drugs that can block ion channels. In addition to inhibiting SERT, CMI inhibits the voltage-dependent Ca^2+^, K^+^, and Na^+^ channels in neuronal and myocardial cells [20,27,28]. Spermatogonia may contain voltage-dependent channels that are affected by CMI. In addition, in the first days after birth, these cells are characterized by marked mitotic activity [17] that affects spermatogenesis in adulthood.

Studies of human [29], hamster, horse, and rat spermatozoa [12] have found the presence of serotonergic markers (5-HT, TPH1, MAOA, 5-HT1B, 5-HT2A, 5-HT3, SERT) and TPH enzymatic activity. This has led to the proposal that 5-HT stimulates PLC/IP3 signals through the 5-HT2A receptor and tmAC/PKA/CatSper channel signals through the 5-HT4 receptor to induce this reaction [30,31,32,33,34]. In adult rats, sperm hyperactivation is enhanced after these stimulations activate sAC and PKA [35]. CMI acts by preferentially inhibiting SERT. The existence of 5-HT and/or serotonergic receptors and SERT in rat sperm and epididymides suggests that neonatal CMI treatment modifies the long-term response of 5-HT and its receptors.

Sperm cells are a key target in studies on male reproductive toxicity designed to assess the processes of spermatogenesis and fertility. Previous studies have documented that the use of antidepressants in male patients of reproductive age can lead to a significant number of sexual dysfunctions, including decreased libido and fertility [36,37,38] and reduced quantity and quality (DNA integrity, motility) of spermatozoa in the ejaculate [39]. Moreover, CMI treatment in adults with psychiatric disorders can modify sperm volume, motility, and morphology [40,41]. However, this has been poorly studied in adulthood when exposure occurs during critical developmental stages.

Our results show that postnatal treatment (8 to 21 PNDs) with CMI produces a deterioration in sperm viability, concentration, motility, and morphology in adulthood. In this way, exposure of male rats during gestation to FXT (20 mg/kg) through the placenta has been associated with impaired testicular development and spermatogenesis in adulthood [42], while administering 20 mg/kg FXT during lactation (0–21 days of age) reduces all sperm parameters significantly, including count, motility, viability, and normal morphology, coupled with a significant increase in the percentage of sperm with chromatin/DNA damage in mice exposed to FXT, compared to controls [43].

Administering FXT during pregnancy or lactation not only reduces sperm parameters (count, motility, viability) but also affects the Sertoli cells, tubular diameter, and the height of the epithelium of the seminiferous tubules [44,45,46]. Some reports indicate a direct correlation between the number of Sertoli cells and spermatogenesis [46], and between the number of Sertoli cells and the epithelial height and diameter of seminiferous tubules [45,47]. In addition, a decreased population of Sertoli cells can produce shrinkage of the seminiferous tubules [32], and low sperm concentrations are related to the decrease in germ cells induced by FXT and citalopram [48,49]. Fluoxetine, specifically, induces damage in the seminiferous tubules and reduces the number of germ cells due to increased ubiquitin (UCHL1) activity in the tubules. This is associated with a high incidence of germ cell death in rats [50]. Thus, neonatal CMI treatment reduces sperm concentrations in adults, possibly due to its effects on the Sertoli and spermatogonial cells in the seminiferous tubules during spermatogenesis, and on the height of the epithelium and tubule diameter.

SSRIs induce DNA damage and apoptosis. Toffoli et al., 2014 [51] demonstrated that exposure to fluoxetine during gestation and lactation affects the DNA methylation of brain of rats, while Przemyslaw et al. (2021) [52] evaluated the differential toxicity of SSRIs such as amitriptyline, escitalopram, fluoxetine and imipramine in mouse spermatogenic cells. SSRIs were shown to impact the formation of micronuclei and the activation of p53/p21 proteins, where p21 is a protein that plays a key role in the self-renewal and differentiation of testicular stem cells, and p53 the provision of controlled cell division [53], resulting in cell cycle arrest and apoptosis of spermatogenic cells. Postnatal treatment with CMI during the cell proliferation process appears to induce DNA damage in spermatozoids and apoptosis of spermatogonia, like previously used SSRIs, resulting in a reduction in the concentration of spermatozoids.

Results revealed that CMI significantly increased the percentage of abnormal sperm in the caput with a reduction in the cauda, an effect like that reported for the administration of FLX during PND 1–21 in mice [43]. During the first days of postnatal life, especially days 8–20, populations of Leydig progenitor cells, Sertoli cells, and spermatogonia have pronounced mitotic activity that expands along the seminiferous tubules [17]. It is precisely during this stage that CMI was administered (8–21 days) and, although the mechanism of the effect of neonatal administration of CMI on sperm abnormalities remains unknown, it may reflect chromosomal abnormalities during meiosis, especially in primary spermatocytes and spermatids [54] because of the effect of CMI on postnatal testicular development. Otubanjo and Mosuro [55] found that the germ cell mutational activity in sperm correlates with sperm head abnormalities. Abnormalities in the head and tail of sperm reflect point mutations in germ cells that cause alterations in the cell organelles involved in forming these regions [43,56,57].

The mature sperm of all mammalian species produce ROS. However, when generated in excess, ROS can induce damage in the sperm function that involves lipid peroxidation and damage to the DNA and proteins that can affect the sperm’s ability to fertilize an egg. As a result, the spermatozoa lose motility, DNA integrity, and vitality. Mitochondria are one of the main sources of ROS formation in sperm [58]. ROS production involves the escape of electrons from sperm mitochondria caused by various factors that interfere with electron flow along the electron transport chain [29]. Research shows that FLX alters mitochondrial functioning by modulating the activity of respiratory chain components and Krebs cycle enzymes, a process that can induce cell death [29,58,59,60,61,62]. In mitochondria isolated from rat and pig brain and liver tissues, FXT interacted with the lipid bilayer of the membrane to inhibit electron transport and F1F0-ATPase activity [29,58,59], citrate synthase activity in the striatum [60], and mitochondrial functioning [61]. CMI has also been shown to cause changes in various mitochondrial functions, such as inhibiting the activity of mitochondrial complex III, decreasing the potential of the mitochondrial membrane, and causing swelling and vacuolation [62]. CMI led to oxidative stress, inflammation and structural changes in the testis, and a reduction in sperm count and motility [6]. Thus, CMI’s inhibitory effect on electron transport chain complexes in the mitochondria could be due to a direct interaction of this drug with the complexes that inhibit them, leading to increased ROS production, oxidative stress as seen in the case of drugs of the cannabinoid family [63]. The observed sperm abnormalities (reduced motility, increased ROS) in adulthood are likely downstream consequences of this early-life disruption in epididymal 5-HT signaling. This establishes a plausible mechanistic link between neonatal pharmacological intervention, long-term alteration of a local neurotransmitter system in a reproductive organ, and adult reproductive dysfunction.

In summary, this study shifts the paradigm from viewing SSRI effects on reproduction solely as reversible adult phenomena to recognizing their potential as developmental disruptors. We provide pioneering evidence that epididymis and its serotonergic system are key targets for such early-life programming. The persistent, region-specific alteration of 5-HT levels appears to be a central mechanism mediating the long-term detrimental effects on sperm quality, highlighting the critical importance of the neonatal period for the establishment of a functional male reproductive system.

## 4. Materials and Methods

### 4.1. Animals

The rats used as experimental subjects were provided by the vivarium at the Universidad Autónoma Metropolitana. All experiments and procedures were carried out in strict accordance with Mexico’s Official Norm NOM-062-ZOO-1999 [64] for the production, care, and use of laboratory animals, and the National Institute of Health’s Guide for the Care and Use of Laboratory Animals [65]. The experimental protocol was approved by Universidad Autónoma Metropolitana-Iztapalapa Academic Ethics Commission of The Division of Biological and Health Sciences, the Ethics Committee number CECBS23-27.

Experimental animals were obtained from pregnant Wistar rats on day 3 of the pups’ postnatal life. The male pups were randomly cross-fostered to maintain a uniform number in each litter (“*n*” = 6 pups/mother), two litters in total. The female pups were excluded. The experimental animals were kept under standard vivarium conditions, on a reversed light cycle (lights on 21:00 h, off at 9:00 h) at a temperature of 24 ± 1 °C, with food and water available ad libitum. The pups were divided into two groups (“*n*” = 6 male rats per group) and received injections twice a day (9:00, 18:00) from postnatal day 8–21. The treatment group received clomipramine (30 mg/kg 0.1 mL, sc), and the control group only a saline solution.

Dosage, route of administration, and duration of CMI treatment were chosen considering its effectiveness in producing the behavioral and physiological abnormalities required by the study protocol [66,67,68,69,70]. On PND 23, the pups were weaned, housed in groups, and maintained in the vivarium. Once they reached 3 months of postnatal life, the following experiments were performed.

### 4.2. Experimental Design

At 3 months of age, the pups were euthanized and their epididymides extracted and dissected. Spermatozoids were obtained from the tail of the left epididymis to analyze sperm viability, capacitation, mitochondrial activity, and DNA damage. The right epididymis and testis were dissected for evaluation of 5-HT levels using HPLC.

### 4.3. Sperm Viability

At the beginning of the dark phase, between 9:00 and 11:00, the pups from both groups (control, CMI; “*n*” = 6) were euthanized by decapitation under deep anesthesia. The cauda, corpus, and caput sections of the epididymides were segmented. Motile sperm was extracted only from the caudal portion. Dissection scissors were used to make two cuts in the cauda. The tissue was removed and placed in an Eppendorf tube with 1 cc of Biggers–Whitten–Whittingham medium (BWW) [71] (mM: 95 NaCl, 5 KCl, 1.7 CaCl_2_, 1.1 KH_2_PO_4_, 1.19 MgSO_4_-7H_2_O, 25.07 NaHCO_3_, 10 HEPES, and 5.56 D-glucose; 37 °C, pH 7.2; Sigma-Aldrich, Saint Louis, MA, USA). Sperm viability was assessed by eosin-nigrosin (1:1) cell staining on slides. At least 100 sperm were counted to discriminate alive from dead units under light microscopy with a 40× objective (Optisum, Long Island City, NY, USA).

### 4.4. Sperm Capacitation

The spermatozoa were put in a capacitation medium (94.6 NaCl, 25 mM KCl, 1.71 mM CaCl_2_, 1.19 mM MgSO_4_, 1.19 mM KH_2_PO_4_,25 mM NaHCO_3_, 5.56 mM glucose, 10.76 mM sodium lactate, 0.5 mM sodium pyruvate, and 4 mg/mL bovine serum albumin, pH 7.4) and incubated for 6 h at 37 °C with 5% CO_2_. After incubation, the sample was fixed with 4% paraformaldehyde, then 15 µL of CTC solution (20 mM of Tris, 130 mM of NaCl, 5 mM of cysteine and 1.5 mM of CTC, pH 7.8) and 15 µL of DABCO (220 mM of DABCO dissolved in PBS and glycerol at a proportion of 9:1) were added. The sample was observed under epifluorescence microscopy (100× objective). Approximately 100 cells were counted [72].

Net sperm capacitation = (percentage of capacitation at the end of capacitation (6 h) − (percentage of capacitation at time 0)

These parameters were determined using the CTC technique.

### 4.5. Mitochondrial Activity

The MTT test (3-(4, 5-dimethyl thiazolyl-2)-2, 5-diphenyltetrazolium bromide) is a sensitive, accurate measure of cellular metabolic activity. It depends on the reduction of MTT—a yellow, water-soluble tetrazolium dye—to purple-colored formazan crystals, predominantly due to the action of mitochondrial dehydrogenases. Once dissolved in DMSO, the crystals were examined spectrophotometrically (550 nm).

### 4.6. Measurement of ROS Production

Dichlorofluorescin diacetate (DCF) was used in these tests, as described previously [24,25], to measure ROS production by flow cytometry analysis (FACSCalibur: Beckton Dickinson, San Jose, CA, USA). Briefly, samples from each group containing 0.5106 spermatozoa were incubated for 15 min at ambient temperature, under darkness, with 1 mL of physiological solution (NaCl 95 mM, KCl 5 mM, CaCl_2_ 1.7 mM, KH_2_PO_4_) and 50 µL of DCF at 32 µM before centrifugation for 5 min at 1500× *g* force. The resulting pellet was dissolved in 1 mL of PBS and examined using the CELLQUEST program (version 5.1, BD Biosciences, San José, CA, USA).

### 4.7. Analysis of DNA Damage

A sample of sperm in physiological solution was used to assess DNA damage. A smear of the sample was dried at room temperature, fixed in Carnoy’s solution for 24 h, stained with acridine orange for 5 min, and observed under epifluorescence microscopy (100× objective). Approximately 100 cells were counted.

### 4.8. 5-HT Analysis by Reverse-Phase High-Performance Liquid Chromatography (RP-HPLC)

For 5-HT extraction in the testis and caput and cauda of epididymis, 400 μL of buffer containing 5% ascorbic acid, 200 mM sodium phosphate, 2.5 mM L-cysteine, and 2.5 mM EDTA were added. To precipitate the protein, 100 μL of 0.4 M perchloric acid was added, with incubation at 20 °C for 20 min. Then, after centrifugation at 12,000 rpm for 10 min (4 °C), the collected supernatants were filtered by 0.22 μm then used for the evaluation of 5-HT by RP-HPLC in a system that consisted of a PU-2089-plus pump (Jasco, Inc., Easton, MD, USA), an AS-2057-plus autosampler (Jasco, Inc.), and an X-LC™3120FP fluorescence detector (Jasco, Inc.). Chrom-Nav software (version 2.0, Jasco, Inc., USA) was used to control all instruments. Chromatographic runs were performed using a Júpiter C18 column (300 Å, 5 μ, 4.6 × 250 mm, Phenomenex®) at 30 °C. The column was equilibrated with mobile phase A containing 0.1% trifluoracetic acid in water. Then a linear gradient was performed from minute 5 to minute 20 with mobile phase B containing 0.1% trifluoroacetic acid in acetonitrile at a flow rate of 0.8 mL/min. The fluorescence detector was set to a gain of 1000, an attenuation of 32, a response time of 20 s, and 280 nm and 315 nm for excitation and emission, respectively, using 50 μL as the sample injection volume.

### 4.9. Statistical Analysis

The variables evaluated were the effects on sperm quality (alive/dead and concentration) and physiology (capacitation, mitochondrial activity, ROS production) in Wistar rat spermatozoa treated with clomipramine and the levels of 5-HT in the testis and epididymis. The Kolmogorov–Smirnov test was applied to determine the normality of the data, while the Levene test was used to determine the homoscedasticity of variances. For the data that showed normality and homoscedasticity, parametric tests were performed using Student’s *t*-test. The IBM SPSS Statistics v. 24.0.0 program was used.

## 5. Conclusions

This study provides novel evidence that postnatal SSRI exposure has long-term, detrimental effects on male reproductive function in adulthood. Treatment with CMI during a critical postnatal window alters the serotonergic microenvironment of the epididymis in a region-specific manner (reducing 5-HT in the caput while increasing it in the cauda), a finding not previously reported. These persistent changes in epididymal 5-HT are associated with impaired sperm maturation, evidenced by reduced sperm quality and hyperactive mitochondrial function. Our work underscores the epididymis and its local 5-HT system as vulnerable targets for early pharmacological intervention and establishes a new framework for understanding how neonatal exposures can program adult reproductive disorders via disruption of neuroendocrine pathways in reproductive organs. This opens potential future research on the role of 5-HT in testicular and epididymal development, as well as its differential role in different regions of the epididymis. Furthermore, the mechanism by which 5-HT dysfunction is linked to ROS generation should also be analyzed.

## Data Availability

The original contributions presented in this study are included in the article. Further inquiries can be directed to the corresponding author.
